# Levetiracetam as First-Line Anti-seizure Medicine in Electrographically Confirmed Neonatal Seizures: A Literature Review

**DOI:** 10.7759/cureus.101814

**Published:** 2026-01-18

**Authors:** Khalid M Saifullah

**Affiliations:** 1 Pediatrics and Neonatology, International Hospital, Salmiya, KWT

**Keywords:** efficacy, electroencephalogram, levetiracetam, neonatal seizure, phenobarbitone, safety

## Abstract

Phenobarbitone (PHB), the recommended first-line anti-seizure medicine (ASM) in neonatal seizure (NS), has many side effects needing monitoring and management; therefore, it may not be a good choice in low-to-middle-income countries (LMICs) with poor facilities. Levetiracetam (LEV), an emerging ASM in NS with a good short-term safety record, is evaluated with or without a comparator ASM as a first-line ASM. Using strict inclusion and exclusion criteria, a comprehensive literature search was undertaken using PubMed, Medline, Cochrane, CINAHL, Embase, Clinical.Trials, Scopus, Trip database, and NHS Evidence. The quality of the papers was assessed using Critical Appraisal Tool Program (CASP), and the risk of bias (ROB) was assessed using the Newcastle-Ottawa Scale (NOS). Data was extracted in a predefined proforma. In total, five retrospective observational studies were included in the review. All included studies suffered from moderate to high ROB. The evidence suggests that when compared to PHB, LEV is possibly equally effective and safer, and, when used without comparison, is effective in neonates with low seizure burden. However, it cannot be recommended as a first-line ASM in NS due to poor quality of evidence. Well-designed randomized controlled trials are urgently needed, preferably from LMICs.

## Introduction and background

Neonatal seizure (NS) is a serious neurological emergency and, if not promptly identified and treated, can lead to devastating outcomes like death, epilepsy, cerebral palsy (CP), and cognitive dysfunction [1]. The incidence of NS varies in different studies depending on population and diagnostic methods. In term newborns, it is one to four per 1000 live births [2] while in preterm newborns the incidence is much higher at 14 to 40 per 1000 live births [3]. Studies from low-to-middle-income countries (LMICs), where incidence of perinatal asphyxia and hypoxic-ischemic encephalopathy (HIE) is highest, can underestimate the true incidence of NS due to a lack of EEG [4].

The etiology of NS can differ in term and preterm newborns. HIE is the commonest cause in term newborns, responsible for 50%-77% cases, while in preterm newborns, intraventricular hemorrhage (IVH) is the commonest cause [5,6]. Other causes common in both gestational ages are stroke, infection, and genetic, structural, and metabolic causes [5].

One of the major challenges in neonatal neurology is the diagnosis of NS. Traditionally, NS is defined as a paroxysmal alteration of neuronal function resulting in motor, behavioral, or autonomic manifestations [7], but this is difficult to apply in newborns as all epileptic phenomena are not manifested clinically due to immature brain pathways [8]. A study using video EEG (vEEG) found only 34% clinical correlate of EEG seizures in HIE patients and only 27% clinically suspected seizures had EEG confirmation [9]. The recent guidelines by the International League Against Epilepsy (ILAE) classify only EEG-based seizures as confirmed seizures [10]. This poses practical and ethical problems in LMICs where EEG is often unavailable, and the clinicians must start treatment based on clinical suspicion because of the concern for adverse developmental outcomes [11]. Amplitude-integrated EEG (aEEG) is available more commonly in such settings, but its sensitivity is only 50% or 80% (using two or four leads, respectively) compared to continuous EEG (cEEG) [12].

Another major issue related to NS is its treatment. Phenobarbitone (PHB) is the recommended first-line anti-seizure medicine (ASM), both by the World Health Organization (WHO) [13] and the ILAE [10]; however, its success rate in controlling seizures is only 50%-80% [14,15]. Also, PHB has many safety concerns, both short- and long-term. It has shown to cause increased apoptosis in animal brain [16] and synaptic disruption in hypoxia-induced rat brain [17]. Children exposed to PHB during the antenatal period or in childhood may have poor motor and cognitive functions [18,19]. The short-term adverse effects of PHB can be profound, such as hypotension, bradycardia, and respiratory depression, mandating availability of monitors and ventilators to manage such cases [20,21]. Phenytoin (PHE) and benzodiazepines (BZD) are the other commonly used first-line ASMs but have similar limitations as PHB in causing cardio-respiratory depression and adverse long-term neurodevelopmental outcome (NDO) [4,14]. These concerns have led clinicians to search for safer and possibly more effective first-line ASMs.

Levetiracetam (LEV), commonly used in adult and pediatric epilepsies, received US Food and Drug Administration (FDA) approval in 2012 for use in infants above one month and since then, its off-label use in newborns has increased in some regions [22]. LEV is postulated to have a unique mechanism of action. It is a pyrrolidine derivative and binds to a synaptic vesicle glycoprotein SV2A in rapidly firing neurons, inhibiting presynaptic neurotransmitter release [23]. What may make LEV a safer ASM is its pharmacokinetic profile, which has been studied in term newborns [24]. Unlike PHB and PHE, it has linear pharmacokinetics with complete oral absorption, renal clearance which matures with gestational age, no hepatic metabolism, and minimum drug-to-drug interaction; therefore, not needing therapeutic drug monitoring [25]. Its pharmacokinetics in preterm newborns, though not separately studied, caused fewer adverse effects compared to PHB in a few studies [26,27].

The safety data of LEV has been accumulating in recent years through several animal and human studies. When compared with other commonly used drugs in NS, it caused less synaptic disruption [28] and less apoptosis in hypoxia-induced rat brains [17]. A follow-up study at 24 months in clinically diagnosed NS cases treated with either PHB or LEV found increased incidence of motor and cognitive dysfunction with PHB compared to LEV (p=0.01) [29]. Another short-term follow-up study at one month in 38 infants with EEG-confirmed seizure cases who received either PHB or LEV found better tone and posture in LEV-exposed infants [30].

Unlike safety, the efficacy of LEV as a first-line ASM is not yet proven through studies in various settings. The ILAE [10] recommends it as a second-line ASM based on expert opinion. So far, no study that diagnosed seizures on EEG has found LEV superior to PHB, while many studies with clinical diagnosis of seizure found it equally effective or superior to PHB [27,31,32]. To date, the only methodologically sound, multi-centric randomized controlled trial (RCT) [15] found PHB significantly superior to LEV in controlling seizure (80% versus 22%, p<0.001); however, on increasing the dose of LEV from 40 mg/kg to 60 mg/kg, there was 7.5% more control in seizure, prompting the authors to suggest using higher doses in future trials.

Few systematic reviews (SRs) and meta-analyses (MAs) are available (Table 1) on the efficacy and safety of LEV; however, their methodological limitations prevent us from drawing any firm conclusion about the first-line ASM in NS [20,21,33,34]. Major limitations of these reviews are clinical diagnosis of seizures, variable doses of LEV (10-100 mg/kg), its use as a second- or third-line ASM, and treatment response assessment either clinically or on EEG. The evidence from these studies suggests LEV to be equally effective and safer than PHB. The only review [33] that used EEG-based seizure diagnosis in its inclusion criteria selected most of the studies with very small (n=3-4) populations who received LEV, some using LEV as second-line ASM, and compared LEV patients with historical cohorts of PHB. The pooled effect of LEV as primary ASM in controlling seizure was 77% (37/48) compared to 46% (24/52) by PHB (‘p’ value not given). No adverse effect of LEV was reported, raising doubt about publication bias.

**Table 1:** Summary of Published Systematic Reviews on Efficacy and Safety of Levetiracetam (LEV) NS: neonatal seizure, RCT: randomized controlled trial, PHB: phenobarbitone, ASM: anti-seizure medicine, MA: meta-analysis

Authors	Population	Study design	NS Diagnosis	LEV use	Main Outcomes	Limitations
McHugh et al. [33]	Five studies- 102 cases (term and preterm)	Retrospective	EEG-based	As first-line, second-line, and adjunctive	LEV equally or more effective than PHB when used as primary ASM (77% VS 46%, Significance value not given). Adverse effects not reported	-Historical control of PHB -Secondary and adjunctive use of LEV -Very small sample size (3-4 LEV cases) in few included studies -Great heterogeneity in loading dose of LEV (5-50 mg/kg) -Studies included till 2013 when LEV use was uncommon -Publication bias may not be ruled out
Hooper et al. [21]	14 studies, 1188 cases (term and preterm)	RCT and observational	Clinical and EEG	As first-line	-Pooled efficacy from observational studies -LEV efficacy 45% (95% CI 34-57%) -MA of RCTs -LEV and PHB equally effective (RR 0.6 95% CI 0.3-1.2) -Short-term adverse effect low in LEV as compared with PHB (RR 0.24, 95% CI 0.06-0.92)	-Most studies had clinically diagnosed NS -Seven observational studies with no control arm -Certainty of evidence rated very low
Qiao et al. [20]	Term and preterm, 24 studies	RCT and observational	Clinical and EEG	As first-line, second-line, and third-line	-No significant difference in efficacy between LEV and PHB (OR 0.79, 95% CI 0.25-2.44) -Higher adverse effects with PHB (OR 5.61, 95% CI 2.53-12.44)	-Most studies included clinical seizure -LEV use as second- and third-line -Heterogeneity in included studies (I^2^ 85%)
Kumar et al. [34]	10 studies, 786 cases (term and preterm)	RCTs	Clinical and EEG-based	LEV as first-line	-LEV non-inferior to PHB (RR 1.11, 95% CI 0.79-1.54, I2 86%) -LEV resulted in fewer adverse effects	-Clinical diagnosis of NS -Very low quality of evidence -High heterogeneity in studies

From this it can be seen that these SRs suffer from many methodological limitations: clinical diagnosis of NS, use of LEV as second-line ASM, heterogeneity in doses, and when LEV was shown to be more efficacious and safer, the quality of evidence in the selected studies was rated very low. The only study that selected EEG-based seizures [33] included literature till 2013, a time when LEV use was uncommon; also, comparison with PHB was from historical controls and most of the selected studies were case reports using LEV in three to four patients only.

Therefore, the choice of ASM in NS is far from established. In the setting of LMICs, the facilities to manage complications of PHB may not be available (monitors and ventilators) and lack of EEG facility in most of the units presents a unique ethical issue of missing NS cases, which if not timely treated can result in adverse outcomes. By relying on clinical features to diagnose NS, non-epileptic phenomena may be wrongly diagnosed as true seizure thus exposing the immature brain to potential toxic effects of ASM. Therefore, the choice of ASM should be based on a confirmed diagnosis of NS and, where facilities for such confirmation are unavailable, the efficacy and safety of the chosen ASM should be within an acceptable range. To resolve these issues, a literature review using strict criteria of EEG-based seizure diagnosis and comparison of LEV as a first-line drug with other first-line ASMs was urgently needed. A step-by-step approach based on evidence-based practice is discussed in the following paragraphs to carry out this review.

## Review

Methods

Framing the Research Question

A common tool for framing the research question and identifying the key terms is PICO which stands for Population, Intervention, Comparison and Outcome [35]. The letter ‘T’ is often added in PICO for the types of studies, then this is called PICOT [36].

To know the efficacy and safety of LEV as a first-line ASM when compared with other first-line ASMs, the review question is framed as the following: “In newborns with EEG confirmed seizures, is LEV as first-line ASM when compared with other first-line ASM safe and effective?”. The components of the review question can be broken into five parts of PICOT (Table 2). 

**Table 2:** Population, Intervention, Comparison, Outcome, and Type (PICOT) NS: neonatal seizure, ASM: anti-seizure medicine

PICOT Element	Description
Population	Newborns (Term and Preterm) with EEG-confirmed NS
Intervention	Levetiracetam as first-line ASM
Comparison	Another ASM as first-line ASM
Outcome	Efficacy and safety
Type of studies	Clinical trials

Strict inclusion criterion of neonatal onset seizure diagnosed by EEG, LEV with or without a comparative drug as first-line ASM, research published in English language and after 2013, and outcome measured as efficacy or safety were applied (Table 3).

**Table 3:** Inclusion and Exclusion Criteria NS: neonatal seizure, RCT: randomized controlled trial, PHB: phenobarbitone, LEV: levetiracetam, BZD: benzodiazepines, ASM: anti-seizure medicine, cEEG: continuous EEG, vEEG: video EEG, aEEG: amplitude-integrated EEG, NDO: neurodevelopmental outcome

Review	Inclusion criteria	Exclusion criteria	Comment
Population	Neonatal age (term- 37+0 weeks till 28 postnatal days, preterm- 24+0 to 36+6 weeks till 44 weeks post-menstrual age)	Post-neonatal age	
Diagnosis	Seizure confirmed by cEEG, vEEG or aEEG	Clinically diagnosed NS	Transient metabolic disturbances (e.g., hypoglycaemia, hypocalcaemia, hypomagnesemia and hypo- or hypernatremia) causing seizure were not considered for exclusion as such cases do not need ASMs
Other factors relevant with population		Major congenital anomalies.	Can potentially affect response to ASMs
Intervention	LEV as first-line ASM, intravenous (IV)/oral, any dose/any duration.	LEV as second- or third-line ASM.	
Comparison	Other ASM as first-line except short-term use of BZD OR no comparative ASM.	Comparative ASM as second-line treatment	Short-term boluses of BZDs for controlling seizure in emergency were acceptable as they have short half-life and are not continued after bolus dose.
Outcome	Efficacy: Cessation of EEG seizure for at least 24 hours after loading or maximum dose of ASM. OR Safety: Short-term: Acute events like apnoea, bradycardia, hypotension, somnolence, need for mechanical ventilation. Long-term: NDO	Recurrence of EEG-diagnosed NS within 24 hours of stopping ASM.	Not all studies report combined outcomes of efficacy, safety and NDO, therefore, any of these outcomes were accepted for inclusion.
Types of studies	RCTs, Observational studies (prospective or retrospective cohorts, case-control and cross-sectional	Case reports, case series, expert opinion, guidelines. Studies included in previous reviews.	If a study was part of a previous review, it was considered for inclusion only if any important aspect was not reviewed previously.
Limits applied in literature search	Human subjects, English language and studies from 2013 and beyond.		Excluding studies in non-English languages due to lack of translators. Studies were selected from 2013 onwards because of LEV's common use in newborns after FDA approval in infants in 2012.

Types of Evidence Needed to Answer Review Question

According to the hierarchical ladder of evidence-based medicine, SRs of RCTs and synthesis of their results into an MA are the best designs to answer an interventional question [37]; however, this review was intended to include original studies that have not been part of previous reviews and not the second-hand analysis of already published reviews. An initial scoping search showed that studies meeting the inclusion criteria were mainly observational; therefore, the results of selected studies are presented in a narrative style, as this review did not have the scope to use statistical synthesis for observational studies [38].

Search Strategy

For each component of PICOT, keywords or medical subject headings (MeSH words) were used. The BOOLEAN characters OR and AND were used to join synonyms and each component of PICO, respectively. Symbol $ was used to maximize search in both UK and American English and symbol * was used where one word ends into different words [36] (e.g. Neonat* can search neonates, neonatal and neonatology). Where combined PICO words could not search enough results, simple search phrases like “neonatal seizure” AND “Levetiracetam” were used (Table 4). Medical databases searched were PubMed, Cochrane Library, Medline, Cumulative Index of Nursing and Allied Health (CINAHL), ClinicalTrials.com, Scopus, Excerpta Medica database (EMBASE), and Trip database. For grey literature, NHS Evidence was searched. A hand search of the bibliographies of selected studies was done for any missing studies. 

**Table 4:** Key and MeSH Words of Different Population, Intervention, Comparison, and Outcome (PICO) Components

Population		Intervention		Comparison		Outcome
“Neonat* seizure*” OR “Neonat* onset seizure*” OR “Epileptic seizure*” OR “EEG-confirmed seizure*” OR “Electrographic seizure*”	A N D	“Levetiracetam” OR “Keppra”	A N D	“Anti-seizure medication*” OR “Anti-epileptic medicine*” OR “Phenobarbitone” OR “Phenytoin” OR “Fosphenytoin” OR “Sodium valproate” OR “Valproic acid” OR “Lignocaine” OR “Lidocaine” OR “Topiramate”	A N D	“Efficacy” OR “Safety” OR “Seizure control” OR “Seizure cessation” OR “Short-term Outcome*” OR “Adverse effect*” OR “Neurodevelopmental outcome*” OR “Developmental outcome*”

Quality Assessment of Selected Studies

Quality assessment of the selected studies was done by using the Critical Appraisal Skills Program (CASP) tool [39] for cohort studies.

For the risk of bias (ROB) assessment, the Newcastle-Ottawa Scale (NOS) for observational studies was used, which assessed bias in patient selection and exposure, confounding factors, and bias in outcome assessment and follow-up. A total score between 1-10 was assigned, with 7-10 as low risk, 4-6 moderate risk, and 1-3 high ROB [40].

Data Extraction

To find relevant information for synthesis and analysis of results, a standardized data extraction form modified from Bettany-Saltikov [36] was used.

Results

Search Results

**Figure 1:**
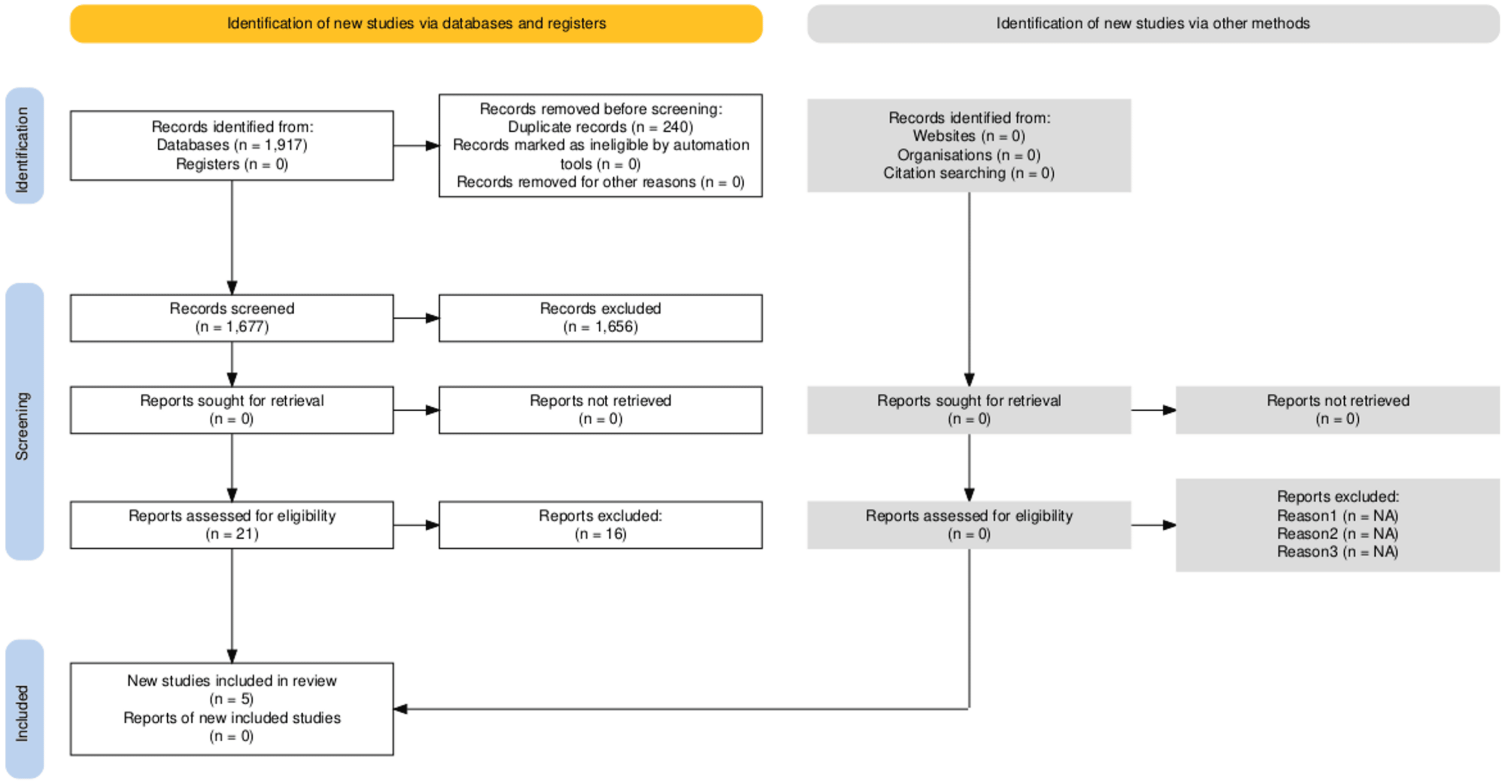
Preferred Reporting of Systematic Reviews and Meta-Analysis (PRISMA) flow for selection of studies

First, all hit articles were screened by their titles. After removing any duplicates, exclusion was made if the study’s aim was different from the review question. Where the title matched the review question, the abstract was read, keeping PICOT inclusion and exclusion criteria. Studies that used clinical criteria for seizure diagnosis, LEV as second- or third-line ASM and those that were already evaluated in previously published SRs were excluded. After abstract screening, 21 studies were shortlisted for full-text reading. Seven ongoing trials’ authors were emailed with a request for the full paper but only one of them replied. Finally, five studies that met the inclusion criteria were selected. The search trail is presented in a Preferred Reporting of Systematic reviews and Meta-Analysis (PRISMA) style (Figure 1) [41].

Analysis of Results

Of the five selected studies (Table 5), four, which are analyzed together, compared efficacy and safety of first-line LEV with first-line PHB, while one study, which was analyzed separately, evaluated the efficacy of LEV as first-line ASM without a comparative first-line ASM in a unique cohort of post-operated congenital heart defect (CHD) patients [42-46].

**Table 5:** Data Extraction HIE: hypoxic-ischemic encephalopathy, CHD: congenital heart defect, ICH: intracranial haemorrhage, PHB: phenobarbitone, LEV: levetiracetam, BZD: benzodiazepines, ASM: anti-seizure medicine, ECMO: extracorporeal membrane oxygenation, IRR: incidence rate ratio, cEEG: continuous EEG, vEEG: video EEG, aEEG: amplitude-integrated EEG

Study no	1	2	3	4	5
Authors	Verwoerd et al. [42]	Keene et al. [43]	Battig et al. [44]	Toptan et al. [45]	Long et al. [46]
Journal	Journal of Child Neurology	The Journal of Paediatric Pharmacology and Therapeutics	Paediatric Neurology	Healthcare	The Journal of Paediatric Pharmacology and Therapeutics
Country	USA	USA	Switzerland	Turkey	USA
Title	Efficacy of levetiracetam and phenobarbitone as first-line treatment for neonatal seizures.	Retrospective evaluation of first-line levetiracetam use for neonatal seizures after congenital heart defect repair with or without extracorporeal membrane oxygenation (ECMO).	Levetiracetam versus phenobarbital for neonatal seizures: a retrospective cohort study.	Comparative outcomes of levetiracetam and phenobarbital usage in the treatment of neonatal seizures: a retrospective analysis.	Efficacy of levetiracetam vs phenobarbital as first line therapy for the treatment of neonatal seizures.
Study design	Retrospective observational	Retrospective observational	Retrospective observational	Retrospective observational	Retrospective observational
Population	Term newborns	Term newborns	Term & preterm newborns	Term & preterm newborns	Term newborns
Sample size	25	18	108	104	87
Aetiology of NS	HIE, ICH, stroke, others	Post CHD repair	HIE, vascular, structural, genetic, infection, metabolic, unknown	HIE, vascular, structural, genetic, infection, metabolic, unknown	HIE, ICH, stroke, epilepsy, infection, metabolic, unknown
Diagnosis	vEEG	cEEG	cEEG	cEEG/aEEG	cEEG/aEEG
Exclusion criteria	-Initial treatment with ASM other than LEV or PHB -Treatment before initiation of vEEG	-LEV not used as first-line ASM -Seizure due to acute metabolic derangements	-First line ASM other than LEV or PHB -Seizure unconfirmed by EEG -Refusal of parental consent	-First line ASM other than LEV and PHB -Seizure unconfirmed by EEG	-Age >60 days -Received ASM outside -Seizure unconfirmed by EEG
LEV (loading & cumulative doses, route	-Loading 50 mg -Cumulative 100 mg -IV	-Loading 30 (20-40) -Cumulative 40 (20-40) in non-ECMO, 65 (48-80) in ECMO (p=0.011) -IV	-Loading 30 mg -Cumulative 60 mg -IV	-Loading 30 mg -Cumulative dose 60 mg -IV	-Loading 60 mg -Cumulative up to 120 mg -IV
Comparative first-line ASM (loading & cumulative doses, route)	PHB -Loading 20 mg -IV	None	-PHB loading 20 mg -Cumulative 40 mg -IV	-PHB loading 15-20 mg -Cumulative 40 mg -IV	-PHB Loading 20 mg -Cumulative 30 mg -IV
Outcomes (Efficacy)	Primary outcomes - Sustained seizure burden<10% (6 min/hr)- Total 13/25 (46%); LEV 10/17 (53%), PHB 3/8 (33%), p>.99. - Sustained seizure burden <20% (12 min/hr)- Total 19/25 (76%); LEV 13/17 (76%), PHB 6/8 (75%), p> .99 Secondary outcomes -Sustained seizure freedom after initial treatment- 9/25 (29%); LEV 6/17 (32%), PHB 3/8 (33%), p>.99 -Median absolute change in 1-hour seizure burden (minutes)- Total 15/ 25, -18.3 ( -1-7- 31.9), LEV (total 9/17) -5.3 (-1.2- 27.2, PHB (total 6/8) -22.6 (-4- 35.0), p 0.26 -Treatment with multiple ASMs- total 13/25 (46%); LEV 8/17 (42%), PHB 5/8 (56%), p 0.69 -Median time to seizure freedom (hours)- total 9.2 (0, 16.5); LEV 6.5 (0, 13.5), PHB 30 (6.9, 32.4), p 0.46	-LEV monotherapy resulted in 90% (9/10) seizure control in non-ECMO cohort. -LEV monotherapy resulted in 0% seizure control (0/8) in ECMO cohort (all requiring second-line ASM) (p<0.001) -Time to seizure cessation from first loading dose in non-ECMO vs ECMO cohort was 33 vs 1968 minutes, respectively (p=0.013). -Significant baseline difference between non-ECMO and ECMO cohorts, with later having higher seizure burden, more abnormal neuroimaging findings, received continuous renal replacement therapy (CRRT) and higher cumulative dose of LEV	-Complete seizure response (n=108)- LEV 45% (15/33), PHB 36% (27/75), P=0.40 -Response in acute symptomatic seizure (n= 75)- LEV 45% (9/20), PHB 37% (20/55), P= 0.59 -Response in seizure due to HIE (n=32)- LEV 50% (5/10), PHB 41% (9/22), P=0.71 -Based on generalized linear model the risk for incomplete response to first line ASM was 2.22, 3.26, and 3.53 times higher for occasional seizure (p<0.01), frequent seizures (p<0.001), and status epilepticus (p<0.001) compared with rare seizures	-Of total 104 neonates studied 71 (68.26%) received PHB while 33 (31.74%) received LEV as first-line ASM -Overall response to first-line ASM was 40.38% (40/104). -No significant difference in response rate between PHB (52.53%) and LEV (54.5%), p=0.309 -Incident rate ratio (IRR) indicated that seizure frequency had significant association with response to treatment; lesser response with more seizure frequency; rare seizure IRR 2.09, frequent seizure IRR 3.25, status epilepticus IRR 4.01. -In multivariate analysis seizure response didn’t differ based on gestation and seizure aetiology	-Seizure cessation efficacy, PHB 27.78% (15/54), LEV 27.27% (9/33), p=0.959 -Prior treatment with BZD, PHB- no effect on seizure resolution rate (p=0.733), LEV- significant reduction in seizure resolution (p=0.021).
Outcome (Safety)	Short-term: LEV- none Adverse effect with PHB- one patient had somnolence Long-term outcome not studied	Short-term: No evidence of hypotension following LEV treatment Long-term: Not studied	Short-term adverse effects (hypotension, respiratory suppression and sedation)- PHB 29.33% (22/75), LEV 3.03% (1/33), P<0.001 -Long-term outcome not studied.	Short term adverse effects (hypotension, bradycardia, increased respiratory support, sedation and irritability)- PHB 26.76 % (19/71), LEV 6.06 % (2/33), P<0.001 -Discharge rate- PHB 67.61%, (48/71), LEV 75.76% (25/33), P=0.674 -Mortality rate- PHB 22.54% (16/71), LEV 45.45% (15/33), P=0.045. -Long-term outcome not studied.	Short term- adverse effects (apnoea or hypotension or respiratory depression) -PHB 55.56% (30/54), LEV 0%, p<0.001 -Long-term outcome not studied

Efficacy of LEV When Compared With PHB

All four studies in this group [42,44-46] were retrospective observational which is considered a very low level of evidence for an interventional trial [37]. Several biases in case selection, treatment allocation, outcome assessment, and reporting occurred and compromised the internal validity of the studies. All studies scored moderate to high ROB on NOS.

Population

Of the total 519 NS cases, 195 (37.5%) were excluded for not meeting inclusion criteria, parental refusal, or unknown reasons. Selection bias is possible as the excluded cases could have different underlying characteristics thus responding differently to ASMs. Sample size varied from 25 to 108. Majority (62%) of selected cases were term newborns with gestational age ranging from 26 to 44 weeks. The underlying etiologies for seizure were HIE (34.65%), intracranial haemorrhage (ICH) or stroke (30.76%), genetic or structural (12.70%), infection (6.35%), and unknown (10.70%). Both the groups were evenly matched for underlying etiologies except in one study [46] which had more cases of ICH in the PHB group (p=0.037).

Diagnosis of NS

Seizure was confirmed by EEG in all the studies; however, only one study [42] used vEEG with records evaluated by certified epileptologist thus reducing assessment and outcome reporting bias. Other studies [44-46] used either cEEG or aEEG, depending on the availability of machine or choice of clinicians, also, these authors did not mention the person who reviewed the EEG data. vEEG is recommended as the gold-standard for NS diagnosis as it can correlate clinical seizure with EEG changes and timing of ASM administration with seizure response [10]. Because of the poor sensitivity of aEEG to pick all seizures [12], assessment bias could have occurred in reporting the events before and after ASM. Two studies [45,46] reported the response to ASM on clinical features also, therefore causing outcome assessment bias.

Dose of ASM

The choice and doses of ASMs were not standardized. The loading and cumulative doses of LEV and PHB were 30-60 mg/kg, 60-120 mg/kg and 20 mg/kg, 20-40 mg/kg, respectively. Clinicians could have selected PHB or higher dose of LEV in patients with more frequent seizures, thus introducing performance bias.

Outcome Assessment 

The primary outcome was complete seizure freedom after the loading dose [44-46] or <10% reduction (minutes/hour) in sustained seizure burden [42]. The later outcome though not meeting the efficacy definition of the present review, is more closely associated with NDO [47]. Among the authors who assessed complete seizure freedom, only one [46] mentioned seizure free period of 24 hours; others are unclear about seizure-free duration, which compromised objectivity in outcome assessment.

The other major outcome was rates of adverse effects (bradycardia, hypotension, apnea, respiratory depression, sedation and agitation) between the two ASMs. Authors did not mention using supportive treatments like inotropes and sedatives (likely to be used in HIE patients), which can mask the side effects of ASM.

Statistics

For binary variables, Fisher exact test and X2 test was used, giving results in numbers and percentage. For continuous variables, Wilcoxon 2-square test and Mann-Whitney test were used, and results were presented as interquartile range or mean±standard deviation. Demographic and clinical characteristics were evenly matched between the two groups. Two studies [44,45] used generalized linear regression model to see the effect of gestation, etiology, and seizure frequency on response to ASM. One study [42] had very small population (n=25) for such analysis but another [46], despite an adequate sample size (n=87), did not perform a regression analysis. The significance level was given as ‘p’ <0.05 but no study presented the results with confidence intervals (CI) thus lacking precision in reporting.

Ethics

Except one study [46], authors of all other studies took institutional ethics committee (IEC) approval before conducting the studies. Despite retrospective design, IEC approval is still recommended to maintain confidentiality, as the initial consent for treatment did not mean using patients’ data for research purpose [48].

Results

Of the total 324 NS patients selected for treatment, 116 (35.80%) received LEV and 208 (64.19%) received PHB. There was no significant difference between the two drugs for seizure control (LEV 46.68%, PHB 34.13%, p>0.05). On subgroup analysis [44,45] for effects of gestation, etiology and frequency of seizures, on response to ASM, there was no difference between the two drugs, however, overall response to ASM was deceased in patients with frequent seizure or status epilepticus as compared to rare seizures (p<0.001). One study [42] did a subgroup analysis for higher dose (100 mg/kg) of LEV compared to low dose (50 mg/kg) and found better seizure control (p<0.04) with higher dose. The hospital discharge and mortality rates in two groups were compared in one study [45], while discharge rate was similar (LEV 75.76% vs PHB 67.61% p=0.674), mortality was significantly higher in LEV group (LEV 45.45% vs PHB 22.54%, p=0.045), which is unexplained as both groups had similar baseline characters. Analysis for the effect of prior BZD use on response to ASM was unique feature of one study [46] which showed no effect on efficacy of PHB (p=0.674) but significantly decreased response to LEV (p=0.021).

Three studies [44-46] comparing adverse effects rates in two groups found significantly decreased adverse effects (hypotension, bradycardia, and respiratory depression) in the LEV group compared to the PHB group (0%-6.06% vs 22%-55.55%, p<0.001). One study [42] did not report any significant adverse effect in two groups due to small (n=25) sample size with only eight patients receiving PHB.

No study followed patients for NDO, which is a major limitation in outcome assessment in seizure cases and use of ASM.

Efficacy of LEV Without a Comparative First-Line ASM

One retrospective observational study [43] after IEC approval evaluated the efficacy (reduction in seizure burden) of LEV as first-line ASM in post-operated CHD patients who were and were not receiving extracorporeal membrane oxygenation (ECMO) therapy. PHB was used as a second-line ASM. There were significant baseline differences between ECMO and non-ECMO groups, with former having more seizure burden (17% vs 1.64%, p=0.003), more abnormal neuroimaging (75% vs 12.5%, p=0.041) and received more continuous renal replacement therapy (CRRT) (63% vs 0%, p=0.007). Seizure was diagnosed by cEEG but records were reviewed by epileptologists at every four-hour intervals or on clinical suspicion that could have missed EEG-only seizures. Efficacy of LEV in the non-ECMO cohort (LEV dose 50 mg/kg) was 90% (9/10), while in the ECMO cohort (LEV dose 65 mg/kg) it was 0% (0/8). Use of PHB was 100% in ECMO cases as compared to only 11.1% in non-ECMO cases (p<0.001). Because of multiple supportive medicines (inotropes and sedatives), the adverse effect of LEV could not be reported. This study suffered from moderate ROB in NOS. These results can be applied in similar settings where LEV can be used as a first-line ASM in hemodynamically stable patients with low seizure burden; however, similar generalization cannot be made in more usual settings of NS.

Discussion

This literature review included five retrospective observational studies evaluating efficacy and safety of first-line LEV in EEG-confirmed NS. Four studies compared LEV with PHB as first-line ASM in usual settings of NS, while one study used LEV as the only first-line ASM in post-CHD repair cases. All studies suffered from moderate to high ROB. The evidence suggests that LEV has equal efficacy and better short-term safety when compared to PHB and can effectively control seizure if used in stable post-CHD repair patients with low seizure burden. LEV was more effective in higher doses [42] and less effective if BZD was given prior to its administration [46]. The comparable efficacy of LEV with PHB was maintained irrespective of gestation, seizure etiology, and frequency of seizure. The results of present review agree with previously published reviews [20,21,33,34]; however, unlike this review, others included studies with clinical diagnosis of seizure and in some LEV was used as second- or third-line ASM. Similar to this review, others also included mostly observational studies, which had variable LEV dosing and different methods of outcome assessment.

Due to the observational design of included studies, this review has several limitations. Selection bias could have occurred, as more serious cases of NS would have received treatment before EEG monitoring or during transfer to the study center. Short-term use of BZD though was not an exclusion criterion in this review, and could have affected the response to PHB and LEV [32,46]. Also, the use of a low dose of LEV in many studies could have resulted in less seizure control [15,42]. Use of aEEG or intermittent review of EEG might have missed EEG-only seizure in some studies. Adverse effects, which were evaluated from patients' records, could have missed some events like sedation and irritability, as observers would regard them as inconsequential. Also, use of sedatives and analgesics which is a common practice in sick newborns (HIE and post-CHD cases), could have masked adverse effects of ASM. Because of small sample size in two studies [42,43], adverse effects were not reported. One study [45] has many inconsistencies in results presentation and interpretation that compromised its internal validity. The study on post-operated CHD patients [43] had highly specialized population, therefore, results cannot be extrapolated in other settings. Other limitations of this review are, single person review which limited cross checking of searched literature and, restricting the studies to English language due to unavailability of interpreter which could have missed studies in other languages thus introducing publication bias. Limited scope of the review precluded statistical synthesis of data; therefore, the results are presented in a narrative style, which gives less objectivity in its interpretation.

Despite these limitations, strict selection criteria, thorough literature search, and a balanced appraisal of each study with ROB assessment are highlights of this review. Authors of the included studies collected data from a real-world scenario where randomization and blinding are often not possible due to highly unpredictable and dynamic nature of patients. The contrasting results between many observational studies and a single well-conducted RCT [15] on therapeutic efficacy of LEV pose a dilemma for treating clinicians, especially in LMIC settings where diagnostic and monitoring facilities for NS may not be available. The recommended first-line PHB may not be a suitable option, especially in HIE cases where avoiding hypotension is a paramount concern; therefore, in such scenarios, LEV in higher doses can be an alternative first-choice ASM.

## Conclusions

This literature review found LEV to be safer and possibly equally effective first-line ASM when compared to PHB. The included studies are of poor quality due to multiple ROB; therefore, a firm conclusion cannot be drawn about LEV's use as a first-line ASM. Treating neonatologists should use their own judgment based on the current evidence and available facilities to manage NS. In low-resource settings where monitoring facilities are unavailable, LEV can be tried in higher doses. Well-conducted RCTs, especially from LMIC settings, are urgently needed.
